# Integration of COVID-19 and TB screening in Kampala, Uganda - Healthcare provider perspectives

**DOI:** 10.21203/rs.3.rs-1448831/v1

**Published:** 2022-06-07

**Authors:** Fred C Semitala, Rodgers Katwesigye, Dennis Kalibbala, Mary Mbuliro, Rejani Opio, Darius Owachi, Edgar Atine, Josephine Nassazi, Stavia Turyahabwe, Moorine Sekadde

**Affiliations:** Makerere University College of Health Sciences; Makerere University Joint AIDS Program; Makerere University Joint AIDS Program; Makerere University Joint AIDS Program; Makerere University Faculty of Medicine: Makerere University College of Health Sciences; Kiruddu National refferal Hospital; Makerere University Joint AIDS Program; Makerere University Joint AIDS Program; Republic of Uganda Ministry of Health; Republic of Uganda Ministry of Health

**Keywords:** Tuberculosis, COVID-19, healthcare providers, integrated screening

## Abstract

**Background:**

Following the first wave of COVID-19 outbreak, Uganda experienced a 40% drop in Tuberculosis (TB) screening by June 2020. We sought to identify barriers to and facilitators of integrated COVID-19 and TB screening from the perspective of healthcare providers (HCP) at a National Referral Hospital in Kampala, Uganda.

**Design/Methods:**

We conducted a cross sectional study using in-depth interviews with 12 HCP involved in TB activities in the outpatient and emergency departments at Kiruddu National Referral hospital Kampala, Uganda. We explored the HCP experiences at work in the setting of COVID-19, HCP perceived effect of COVID-19 on TB screening activities at the Hospital, and perceptions about social and contextual factors that might influence the willingness of HCP to integrate screening of COVID-19 and TB. We analyzed the data using an inductive thematic approach and the emergent themes were denoted as barriers to and facilitators of COVID-19-TB integrated screening. We then mapped the themes to the Capability, Opportunity, Motivation and Behavior (COM-B) model.

**Results:**

The facilitators to integrated COVID-19 and TB screening included; HCP knowledge of how to separately screen for TB and COVID-19, availability of TB focal persons and interest in learning how to provide integrated screening for TB and COVID-19. The barriers included; HCP inadequate knowledge on how to integrate screening of TB and COVID-19, absence of simple standard operating procedures and data collection tools for integrated screening, inconsistent supply of personal protective equipment (PPE), under staffing, and fear of contracting COVID-19 infection. The identified intervention functions to address the facilitators or barriers included education, persuasion, enablement, and training.

**Conclusions:**

These findings provide a basis for designing contextually appropriate interventions targeting factors that are likely to influence HCP decisions and willingness to conduct TB screening in the context of COVID-19.

## Background

Coronavirus disease 2019 (COVID-19) is caused by the severe acute respiratory syndrome coronavirus 2 (SARS-CoV-2) infection and is a disease of global concern since early 2020. In March 2020, the World Health Organization (WHO) declared COVID-19 a global pandemic[1]. Since then, the global and local response to COVID-19 has caused severe disruptions to the service delivery for other diseases including Tuberculosis (TB) [2–6]. The commonest symptoms of TB (fever and cough) are similar to those exhibited by COVID-19 patients[7], which may negatively affect people’s health-seeking behavior for fear of stigmatization[8, 9]. This may also make healthcare providers less receptive of patients presenting with COVID-19 like symptoms from other causes such as TB[10].

**Figure 1:**
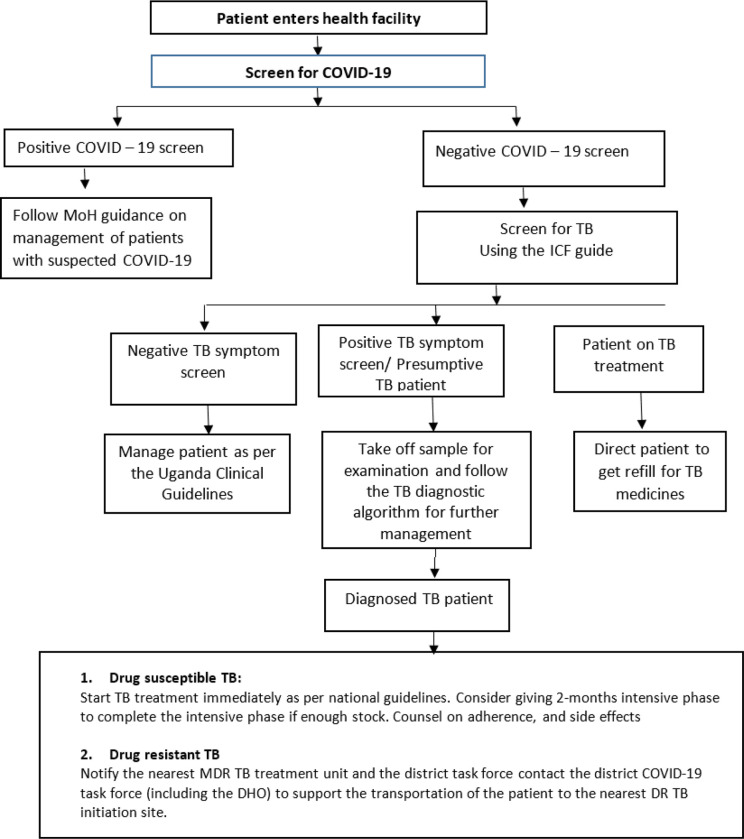
Health facility Tuberculosis (TB) Management Plan in the Context of COVID-19 guidance by Uganda Ministry of Health (MoH); DHO District Health Officer; ICF- Intensified Case Finding

Following the COVID-19 outbreak, like has been documented elsewhere [9, 11, 12], Uganda experienced a 40% drop in TB screening by June 2020; based on the unpublished Ministry of Health programmatic data from the District Health information system version 2 (DHIS2)[13]. In response to this very significant drop in screening for TB, the National TB and Leprosy Program (NTLP) at the Ministry of Health (MoH) developed a health facility TB management plan in the context of COVID-19. The plan included the development of an algorithm to integrate screening for COVID-19 and TB (COVID-19-TB screening algorithm) (Fig. 1). Using this algorithm, ret all patients who present to the health facility should be screened for COVID-19. For patients who screen positive for COVID-19, the MoH guidance on management of COVID-19 suspects should be followed. Patients who screen negative for COVID-19 should subsequently undergo TB symptom screening using the TB intensified case finding guide. Patients with a positive TB symptom screen (presumptive TB cases) should be followed by confirmatory testing with Gene Xpert MTB/RIF (Xpert; Cepheid, USA).

The aim of this qualitative study was to identify facilitators of and barriers to integrated COVID-19 and TB screening from the perspective of HCP at a Referral Hospital in Kampala, Uganda and map these onto the COM-B model and Behavior Change Wheel (BCW) framework to identify suitable intervention functions. We utilized the Capability, Opportunity, Motivation - Behavior (COM-B) model to explore facilitators of and barriers to integrating COVID-19 and TB screening in behavioral terms. We chose the COM-B model, which forms part of the Behavior Change Wheel framework, to understand the capabilities, opportunities and motivations for HCP to integrate screening of COVID-19 and TB.

**Figure 2:**
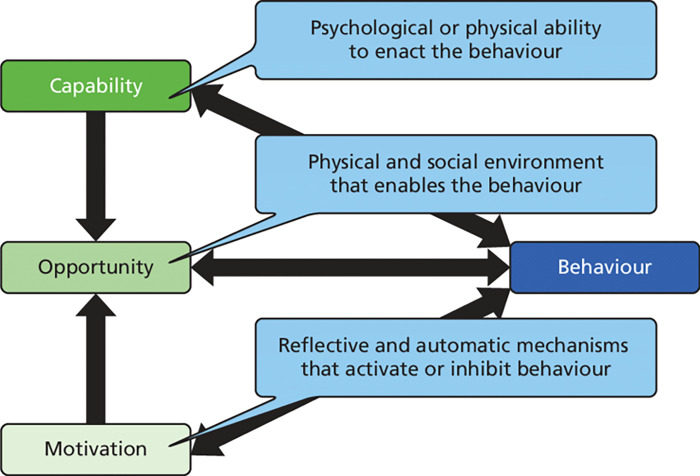
The COM-B model of Behavior. Adapted from the original figure (Michie et al.) [8]

The central principle for the COM-B is that changing any behavior requires changing capability, opportunity and/or motivation to perform that behavior [14]. Thus, the COM-B model (Fig. 2) provides a coherent basis for exploring barriers to and facilitators [15]. This behavioral analysis based on the COM-B would inform the necessary interventions that need to be implemented to promote integrated screening of COVID-19 and TB amongst HCP.

The goal was to inform the design of a contextually appropriate strategy to integrate screening of COVID-19 and TB in Uganda.

## Methods

### Study design and setting

We conducted in-depth interviews between January 2021 and March 2021 with HCP involved in TB-related care activities (TB screening and treatment) at Kiruddu National Referral Hospital (KNRH) in Kampala, Uganda. KNRH is a 200-bed capacity hospital in Makindye Division, one of the five administrative units of Kampala; the Capital City of Uganda. The hospital specializes in Internal Medicine, burns and plastic surgery and provides up to 500 outpatient consultations daily.

We used the Consolidated Criteria for Reporting Qualitative Research (COREQ) guidelines in reporting this qualitative study[16] (see Additional file 1). The study received ethical approvals from the AIDS support organization (TASO) research and Ethics Committee **(TASO REC 082/2020-UG-REC-009)** and the Uganda National Council of Science and Technology **(HS1152ES).**

### Study participants and sampling

We purposively sampled HCP involved in TB-related care activities in the outpatient and emergency departments at KNRH. The maximum sample of HCP was determined by data saturation as proposed by Lincoln and Guba[17].

We recruited different cadres of eligible HCP including medical officers, nurses and TB community linkages facilitators. While observing the COVID-19 prevention guidelines we approached HCP face to face and we informed all of them that the goal of the study was to integrate screening for COVID-19 and TB.

### Study instruments and data collection

We developed interview guides with open-ended questions, designed to explore barriers to and facilitators of integrated COVID-19 and TB screening as perceived by HCP involved in TB-related care activities at KNRH. These included questions about HCP work experience in the setting of COVID-19 (i.e. how COVID-19 activities had affected routine TB screening), HCP perspectives regarding integration of COVID-19 and TB screening (i.e. their thoughts on its importance, how it could be achieved at their departments, factors that could influence their decisions to accept it or not, concerns and likely challenges to the acceptability, feasibility and scaling up the use of the COVID19-TB Screening Algorithm).

The interview guide was drafted in English, piloted and refined using a convenience sample of HCP at the hospital who were not participating in the study. Interviews with HCP lasted between 25 to 40 minutes. Informed verbal consent was obtained from all participants. All interviews including the informed verbal consent were audio-recorded and transcribed verbatim.

All transcripts were de-identified and stored in a secure digital folder accessible only by the research team.

### Research team

All interviews were conducted at the Kiruddu National Referral Hospital, a setting familiar to study participants, by the local study team. The team comprised of social scientists, Implementation scientists, physicians, National TB policy implementers and other health scientists. A Masters-trained social scientist, (DK, male), trained the team before the data collection and supervised the data collection process. A bachelor’s trained social scientist (MM, female) conducted the initial interviews with health care providers while the study nurse (JN, female) attended the sessions to take notes. The social scientist and the study nurse conducted subsequent interviews with Healthcare providers. The interviewers did not know the study participants prior to study commencement.

### Data analysis

Four members of the research team (FCS, RK, DK and DO) analyzed the data using a thematic approach. We preferred thematic analysis because it is suitable for examining the perspectives of different research participants, highlighting similarities and differences, and generating unanticipated insights [14, 16] We adopted an inductive approach using open coding that facilitated the identification of themes in the data. Initially, two analysts (RK, DK) read three similar transcripts independently familiarizing themselves with the data and documenting thoughts on potential codes and themes and then exported the transcriptions to Atlas.ti version 8. The team then met to debrief and compare the initial codes generated by each analyst. We discussed Coding discrepancies and resolved with the other members of the research team (FCS and DO). We developed a coding framework and applied it to the remaining transcripts. We noted the themes emerging during the coding processes and reviewed them in subsequent team meetings where we discussed and developed a consensus on the themes documented.

We categorized the emergent themes as either potential facilitators or barriers to integrating COVID-19 and TB screening. Themes that positively influenced integrating COVID-19 and TB screening were denoted facilitators, and those that negatively influenced integrating COVID-19 and TB screening were denoted barriers. We extracted specific quotations from the transcripts to illustrate verbatim expressions of matters that appeared important.

We developed a behavioral diagnosis by mapping the emergent facilitators and barriers onto their associated COM-B domains.

We then used the Behavioral Change Wheel (BCW) framework to identify potential interventions to promote the facilitators of and overcome the barriers to integrating COVID-19 and TB screening.

## Results

### Demographic characteristics of study participants

Twelve healthcare providers participated in the interviews. They included seven medical doctors, three nurses and two community linkages facilitators. Six healthcare providers (50%) were female. The duration in service at the current post ranged from 3 months to 8 years (median 2.25 years; IQR: 1.5 – 4 years).

### Healthcare provider-reported facilitators of integrating COVID-19 and TB screening

Most HCP reported having knowledge of how to screen for TB and COVID-19 as independent diseases.

“My experience in screening TB in the presence of COVID-19 is that some times you may not be sure whether you are dealing with TB or COVID-19. Because the presentations are a bit similar…but where we have some doubts, we have been referring those clients for COVID-19 testing.”

(Nurse at the hospital)

“So, of course COVID-19 has masked the TB and screening of TB. People have now put much emphasis on COVID-19 leaving out the TB. So we have seen some decline in the cases of TB cases. But it does not mean that these cases have gone down, But because people have put much emphasis on COVID-19 leaving out TB.”

(Medical Doctor at the hospital)

Most healthcare providers reported that TB focal persons were available to support HCP to provide integrated screening for TB and COVID-19.

“… Just like we have the TB focal persons that are already in existence. We have focal persons at the facility level, sub-health district, district and the rest. So, having the focal persons take up another responsibility could also be another key issue for implementation because you may tell people; you conduct your workshop then you leave.”

(Medical Doctor at the hospital)

Healthcare providers also mentioned that having training, mentorships and workshops will help build the capacity for people to understand the relevance of integrated COVID-19 and TB screening.

“The most important thing is knowledge, so you have to give out knowledge. Dispense knowledge to the health workers which means you will have to have a lot of workshops or maybe mentorships throughout the country and training for all the health workers to do the activity.”

(Medical Doctor at the hospital)

### Healthcare provider-reported barriers to integrating COVID-19 and TB screening

Most HCP reported a lack of simple Standard operating procedures and data collection tools to integrate screening of TB and COVID-19. Healthcare provider-reported barriers to and facilitators of integrating COVID-19 and TB screening are summarized in Table 1.

“…So, you find depending on the workload of the staff. if you have a very tedious tool for screening, they may not do it because it consumes a lot of time. But if it is a simplified tool then it can be well utilized, it is easy to maneuver through.”

(Medical Doctor at the hospital)

The HCP also reported an inconsistent supply of personal protective Equipment (PPE) as a constraint in integrating COVID-19 and TB screening.

“If it can be facilitated, it is better but also how long that is not sustainable. If the government can equip the hospital with SOP equipment like sanitizers, temperature guns, like PPE, gloves, cotton. I think it would help health workers accept because they will know that at least our health is well protected”

(Nurse at the hospital)

HCP also reported inadequate staffing levels, coupled with very busy outpatient and emergency departments at the hospital as a hindrance to integrated screening of COVID-19 and TB.

“One of the concerns is the heavy workload because at the end of the day, doing two things at ago while in PPE and you find that it’s one person who is at the unit to do the screening of several patients.”

(Medical Doctor at the hospital)

“We are still understaffed in most places because you have two nurses treating patients on the whole floor or level and yet they want to remove one nurse and take her somewhere else.”

(Medical Doctor at the hospital)

Most HCP feared contracting COVID-19 infection during integrated screening of COVID-19 and TB.

“Anything to do with COVID-19, I don’t want to know, even the ones we are working with [health workers], if she reads a file and sees the word COVID-19 anywhere, that patient will not be seen, treatment will not be given. For me with COVID-19, this government does not care for people, doesn’t care for health workers in case you fall a victim.”

(Nurse at the hospital)

Most healthcare providers also raised the concern of unclear compensation for health workers who contract covid-19 while on duty. They added that their safety is a concern because they are likely to be infected with COVID-19 yet they won’t be compensated.

“.Of course, people need allowances, without allowances they are not going to work [screen for COVID-19 and TB], actually for us we don’t have COVID-19 allowances at this hospital, because they say we don’t treat COVID-19 because COVID-19 is in the communities, yet we treat COVID-19 here. That’s one of the factors that limits health worker involvement in screening for COVID-19. Screening for both is okay, but for COVID-19, most health workers are not interested.”

(Medical Doctor at the hospital).

### Behavioral Diagnosis and Intervention functions

We categorized the reported barriers and facilitators of integrated COVID-19 and TB screening within the domains of the COM-B model to obtain the behavioral diagnosis. For example, identified barriers including; Lack of simple Standard operating procedures for integrated screening of TB and COVID-19, Inconsistent supply of personal protective Equipment (PPE) and Understaffing at the outpatient and emergency departments were mapped to the physical opportunity construct of the COM-B model. Table 2 summarizes the HCP reported facilitators and barriers expressed in terms of their behavioral determinants within the COM-B model.

We linked the behavioral diagnosis obtained using the COM-B model (Table 2) to the behavioral change wheel (BCW) framework and identified appropriate potential interventions functions that could serve to address the reported barriers and facilitators and thereby enhance the acceptance of integrating screening of COVID-19 and TB. These are summarized in Table 3 and Table 4. For example, HCP reported inadequate knowledge on how to integrate screening of TB and COVID-19. Using ***education and training*** as intervention functions, HCP can be equipped with the necessary knowledge and skills through training sessions. Similarly, using ***enablement*** as an intervention function, HCP can be provided simple standard operating procedures for integrated screening of TB and COVID-19, provided adequate supply of PPE and improving the staffing levels in these departments as enablers to facilitate integrated screening for TB and COVID-19.

## Discussion

In this formative cross-sectional study, we explored HCP work experience in the setting of COVID-19, perceived effect of COVID-19 on TB screening and perceptions about social and contextual factors that might influence their willingness to screen for both diseases at Kiruddu National Referral hospital Kampala, Uganda. We utilized the COM-B model to explore barriers to and facilitators of integrating COVID-19 and TB screening[15].

We found that COVID-19 was a real threat to TB related services since HCP at this large hospital were not very well prepared to integrate TB and COVID-19 services at the time. The key potential barriers to integrating COVID-19 and TB screening included; Lack of simple standard operating procedures for integrated screening of TB and COVID-19, Lack of consistent supply of personal protective Equipment (PPE), Understaffing and HCP fear of contracting COVID-19 infection. The key potential facilitators for integrating COVID-19 and TB screening included; availability of TB focal persons to support healthcare providers to provide integrated screening for TB and COVID-19 and their interest and goodwill to be supported and trained to provide integrated screening for TB and COVID-19.

Most HCP had knowledge of how to screen for TB and COVID-19 however; they also reported lack of simple tools such as Standard operating procedures, screening algorithms and data collection tools to support integrated screening of TB and COVID-19. Simple tools such as protocols have been shown to facilitate integration of different programs in clinical settings[18].

Generally, the HCP reported lack of consistent supply of PPE and most of them feared contracting the COVID-19 infection if they were to provide integrated screening for COVID-19 and TB. These fears expressed by the HCPs are consistent with what has been reported from studies in other settings [19–21].

Our findings add to the growing evidence showing that countries and communities should design contextually appropriate and stakeholder informed interventions that adapt active case finding for TB during the COVID-19 pandemic for continuity of TB services [22–26]. In a summary of three operation researches conducted in the capital cities of three African countries (Kenya, Zimbabwe and Malawi), to assess whether a real-time monthly surveillance of TB and HIV activities instead of the usual quarterly surveillance might help to counteract the anticipated negative impact on TB and HIV services, Harries et al [27] demonstrated that there was a decline of 31.2%, 45.6% and 40.6% in the numbers of people presenting with presumptive pulmonary TB for investigation in the three respective countries from March 2020–February 2021 compared to the immediate pre COVID-19 period of March 2019–February 2020. Following the institution of measures to improve TB case detection, there was only a 5% increase in the numbers of people presenting with presumptive pulmonary TB for investigation in Kenya while the numbers in Zimbabwe and Malawi remained far below the baseline period. These studies however, do not describe the steps taken to understand the perspectives of key stakeholders such as HCP in this setting and this may have resulted in suboptimal outcomes.

We have utilized the identified facilitators for and barriers to integrated screening of COVID-19 and TB and the BCW intervention functions to develop the following interventions that are currently being implemented. We have trained 37 HCP on the COVID19-TB algorithm through a series of short interactive sessions to close the knowledge and skills gap; we have printed and distributed job aids of COVID-19-TB algorithm and data collection tools at all the screening points to overcome absence of guidelines on how to screen for TB in the context of COVID-19 and absence of tools for data collection; we have procured and appropriately distributed PPE to the health care providers to overcome the lack consistent supply of PPE and fear of contracting COVID-19 infections.

The Strengths of our study is that it utilizes implementation science approaches including stakeholder engagement and the COM-B model; in this formative assessment, we engaged the HCP who are key stakeholders to understand the contextual factors that may affect their willingness to integrate screening of COVID-19 and TB. Several implementation science studies have demonstrated that engaging key stakeholders in the development of interventions leads to buy-in from the stakeholders, fosters ownership of the intervention and the intervention is more likely to be effective when compared to interventions that are designed without key stakeholder input.

By using the widely applied COM-B model, we made a behavioral diagnosis of the possible challenges to implementing integrated screening for COVID-19 and TB. The framework also provides the behavioral change techniques, which we utilized to design contextually appropriate interventions. The findings from our study are from a single urban national referral hospital and some contextual factors may not be generalizable in different settings.

## Conclusions

The findings of our study highlight the barriers to and facilitators for integrated COVID-19 and TB screening and these findings provide a key stakeholder informed basis for designing contextually appropriate interventions targeting factors that are likely to influence HCP decisions and willingness to accept to use of this algorithm for integrated COVID-19 and TB screening in Uganda and other low and middle income countries. Future studies should evaluate the effect of addressing these barriers to integration of COVID-19 and TB as well as the impact of this on TB case finding in high burden TB settings.

## Supplementary Material

Supplement 1

## Figures and Tables

**Table 1:** Healthcare provider-reported facilitators of and barriers to integrated COVID-19 and TB screening at Kiruddu National Referral Hospital Kampala, Uganda.

Potential facilitators	Potential barriers
**Healthcare providers have knowledge of how to screen for TB and COVID-19 as independent diseases** *“My experience in screening TB in the presence of covid-19 some times you could not be sure whether you are dealing with TB or covid-19. Because the presentations are a bit similar...but where we have some doubts, we have been referring those clients for COVID-19 testing.”* **(Nurse at the hospital)**	**Healthcare Providers lacked adequate knowledge on how to integrate screening of TB and COVID-19** *“So of course COVID_19 has masked the TB, screening of TB. people have now put much emphasis on COVID_19 leaving out the TB. So we have seen some decline in the cases of TB cases. But it does not mean that these cases have gone down, But because people have put much emphasis on COVID_19 leaving out TB.”* **(Medical Officer at the hospital)**
**TB focal persons are available to support Healthcare providers to provide integrated screening for TB and COVID-19** *“...just like we have the TB focal persons that are already in existence. We have focal persons at the facility level, sub-health district, district and the rest. So having the focal persons take up another responsibility could also be another key issue for implementation because you may tell people, you conduct your workshop then you leave.”* **(Medical Officer at the hospital)**	**Lack of simple Standard operating procedures for integrated screening of TB and COVID-19** *“...So you find depending on the workload of the staff. If you have a very tedious tool for screening, they may not because it consumes a lot of time. But if it is a simplified tool then it can be well utilized, it is easy to maneuver through.”* **(Medical Officer at the hospital)**
**Healthcare providers are interested in being supported to provide integrated screening for TB and COVID-19** *“The most important thing is knowledge, so you have to give out knowledge. Dispense knowledge to the health workers which means you will have to have a lot of workshops or maybe mentorships throughout the country and training for all the health workers to do the activity.”* **(Medical Officer at the hospital)**	**Lack of consistent supply of personal protective Equipment (PPE)** *“If it can be facilitated, it is better but also how long that is not sustainable. If the government can equip the hospital with SOP equipment like sanitizers, temperature guns, like PPE, gloves, cotton. I think it would help health workers accept because they will know that at least our health is well protected”* **(Nurse at the hospital)**
	**Understaffing at the outpatient and emergency departments** *“We are still understaffed in most places because you have two nurses treating patients on the whole floor or level and yet they want to remove one nurse and take her somewhere else.”* **(Medical Officer at the hospital)**
	**Lack of simple Standard operating procedures for integrated screening of TB and COVID-19** *“...So you find depending on the workload of the staff. If you have a very tedious tool for screening, they may not because it consumes a lot of time. But if it is a simplified tool then it can be well utilized, it is easy to maneuver through.”* **(Medical Officer at the hospital)**
	**Healthcare providers fear of contracting COVID-19 infection during integrated screening** *“Anything to do with COVID-19, I don’t want to know, even the ones we are working with [health workers], if she reads a file and sees the word COVID-19 anywhere, that patient will not be seen, treatment will not be given. Says this government doesn’t care for people, doesn’t care for health workers in case you fall a victim.* **(Nurse at the hospital)**
	**Lack of risk allowance for healthcare providers to conduct integrated screening of TB and COVID-19** *“..of course, people need allowances, without allowances they are not going to …actually for us we don’t have COVID allowances at this hospital, because they say we don’t treat COVID because COVID is in the communities yet we treat COVID here. That one of the factors that limit health workers involved in screening for COVID, screening for both can be there so no problem but COVID mostly health workers are not interested.”* **(Medical Officer at the hospital).**

Abbreviations; HCP: Health care providers, TB- Tuberculosis, PPE- Personal Protective Equipment

**Table 2:** Barriers to and facilitators for integrated COVID-19 and TB screening at Kiruddu National Referral Hospital mapped to the COM-B model

COM-B constructs	Barriers	Facilitators
**Psychological capability**	Healthcare Providers lacked adequate knowledge on how to integrate screening of TB and COVID-19	Healthcare providers have knowledge of how to screen for TB and COVID-19 as independent diseases
**Physical capability**		Healthcare providers have adequate skills to screen for TB and COVID-19
**Physical opportunity**	Lack of simple Standard operating procedures for integrated screening of TB and COVID-19	
	Inconsistent supply of personal protective Equipment (PPE)	
	Understaffing at the outpatient and emergency departments	
	Lack of data collection tools and databases for integrated screening of TB and COVID-19	
**Social opportunity**		TB focal persons are available to support Healthcare providers to provide integrated screening for TB and COVID-19
**Reflective motivation**	Healthcare providers fear of contracting COVID-19 infection during integrated screening	Healthcare providers are interested in being supported to provide integrated screening for TB and COVID-19
**Automatic motivation**	Lack of risk allowance for healthcare providers to conduct integrated screening of TB and COVID-19	

Abbreviations; HCP: Health care providers, TB- Tuberculosis, PPE- Personal Protective Equipment

**Table 3:** Summary of identified facilitators and linked intervention functions

Capability		Opportunity	Motivation	Intervention functions
Psychological	Physical	Social	Reflective	
HCP have knowledge of how to screen for TB and COVID-19 as independent diseases	HCP have adequate skills to screen for TB and COVID-19	Availability of TB focal persons are available to support HCP	HCP interested in being supported to provide integrated screening for TB and COVID-19	
				Education
		**X**	**X**	Persuasion
**X**	**X**	**X**	**X**	Enablement
				Training

**Abbreviations,** HCP: Health care providers, TB- Tuberculosis

**Table 4:** Summary of identified barriers and linked intervention functions

Capability	Opportunity				Motivation		Intervention functions
Psychological	Physical				Reflective	Automatic	
Inadequate knowledge to integrate screening of TB and COVID-19	Lack of SOPs	Lack of consistent supply of (PPE)	Understaffing	Lack of data collection tools	Fear of contracting COVID-19 infection	Lack of risk allowance	
**X**					**X**		Education
					**X**	**X**	Persuasion
	**X**	**X**	**X**	**X**		**X**	Enablement
**X**							Training

**Abbreviations:** PPE-Personal protective equipment, SOPs- Standard Operating Procedures, TB -Tuberculosis

## Abbreviations

BCW: Behavior Change Wheel framework
COM-B: Capability Opportunity Motivation Behavior
COVID-19: Coronavirus disease 2019
HCP: Health Care Providers
KNRH: Kiruddu National Referral Hospital
MoH: Ministry of Health
NTLP: National TB and Leprosy Program
PPE: personal protective equipment
TB: Tuberculosis
WHO: World Health Organization
