# Supplementary material for: Integration of COVID-19 and TB screening in Kampala, Uganda - Healthcare provider perspectives
Source: Res Sq. 2022 Jun 7:rs.3.rs-1448831. Preprint. [Version 1] doi: 10.21203/rs.3.rs-1448831/v1 (PMC9196119; doi:10.21203/rs.3.rs-1448831/v1)
Supplement: Supplement 1 [file COREQCHECKLIST.docx]

**CONSOLIDATED CRITERIA FOR REPORTING QUALITATIVE RESEARCH (COREQ): 32-ITEM CHECKLIST**

| **No. item** | **Guide questions/description** | **Reported on page no.** | **How the manuscript adheres to the reporting standards** |
| --- | --- | --- | --- |
| **Domain 1: Research team and reﬂexivity** | | | |
| *Personal Characteristics* | | | |
| 1. Inter viewer/facilitator | Which author/s conducted the interview or focus group? | 7 | We describe who led the qualitative research team and who conducted the interviews including their credentials, occupation, gender and experience/ training in qualitative research. |
| 2. Credentials | What were the researcher’s credentials? E.g. PhD, MD | 7 |  |
| 3. Occupation | What was their occupation at the time of the study? | 7 |  |
| 4. Gender | Was the researcher male or female? | 7 |  |
| 5. Experience and training | What experience or training did the researcher have? | 7 |  |
| *Relationship with participants* | | | |
| 6. Relationship established | Was a relationship established prior to study commencement? | 8 | We report that the interviewers did not know the study participants prior to study commencement.  We also report that the participants were informed of the goal of this formative study; which was to inform the design of an implementation of integrated COVID-19 and TB screening, which was to be conducted at the same hospital.  We do not describe interviewer characteristics like bias, assumption and others. |
| 7. Participant knowledge of the interviewer | What did the participants know about the researcher? e.g. personal goals, reasons for doing the research | 6 |  |
| 8. Interviewer characteristics | What characteristics were reported about the inter viewer/facilitator? e.g. Bias, assumptions, reasons and interests in the research topic | N/A |  |
| **Domain 2: study design** | | | |
| *Theoretical framework* | | | |
| 9. Methodological orientation and Theory | What methodological orientation was stated to underpin the study? e.g. grounded theory, discourse analysis, ethnography, phenomenology, content analysis | 8-9 | We describe the inductive thematic analysis approach that we used. |
| *Participant selection* | | | |
| 10. Sampling | How were participants selected? e.g. purposive, convenience, consecutive, snowball | 6 | We describe the purposive sampling technique we used to select participants, how we approached the participants and the sample size.  None of the participants refused to participate/ drop out of the study. |
| 11. Method of approach | How were participants approached? e.g. face-to-face, telephone, mail, email | 6 |  |
| 12. Sample size | How many participants were in the study? | 9 |  |
| 13. Non-participation | How many people refused to participate or dropped out? Reasons? | N/A |  |
| *Setting* | | | |
| 14. Setting of data collection | Where was the data collected? e.g. home, clinic, workplace | 6 | We describe where and how interviews were conducted. We also describe the demographic characteristics of the participants in the study results. |
| 15. Presence of non-participants | Was anyone else present besides the participants and researchers? | 7-8 |  |
| 16. Description of sample | What are the important characteristics of the sample? e.g. demographic data, date | 6,9 |  |
| *Data collection* | | | |
| 17. Interview guide | Were questions, prompts, guides provided by the authors? Was it pilot tested? | 7 | We describe the healthcare provider interview guides, the interview topics and questions and how they were piloted.  We also report that all interviews were audio recorded and field notes were taken during interviews.  We report the average duration of interviews (25 – 40 minutes).  We describe how we arrived at data saturation.  There were no repeat interviews and transcripts were not returned to participants for comment and/or correction. |
| 18. Repeat interviews | Were repeat inter views carried out? If yes, how many? | N/A |  |
| 19. Audio/visual recording | Did the research use audio or visual recording to collect the data? | 7 |  |
| 20. Field notes | Were ﬁeld notes made during and/or after the interview or focus group? | 8 |  |
| 21. Duration | What was the duration of the inter views or focus group? | 7 |  |
| 22. Data saturation | Was data saturation discussed? | 6 |  |
| 23. Transcripts returned | Were transcripts returned to participants for comment and/or correction? | N/A |  |
| **Domain 3: analysis and ﬁndings** | | | |
| *Data analysis* | | | |
| 24. Number of data coders | How many data coders coded the data? | 8 | We describe how data was manually coded initially by two analysts, the debrief process with two other members of the study team and how the coding framework was developed.  We describe how themes were derived from the data using an inductive approach.  Participants did not provide feedback on findings |
| 25. Description of the coding tree | Did authors provide a description of the coding tree? | 8 |  |
| 26. Derivation of themes | Were themes identiﬁed in advance or derived from the data? | 8 |  |
| 27. Software | What software, if applicable, was used to manage the data? | 8 |  |
| 28. Participant checking | Did participants provide feedback on the ﬁndings? | N/A |  |
| *Reporting* | | | |
| 29. Quotations presented | Were participant quotations presented to illustrate the themes/ﬁndings? Was each quotation identiﬁed? e.g. participant number | 9-12 | Quotations are presented to substantiate the analytical findings. We believe that the results are clearly presented. |
| 30. Data and ﬁndings consistent | Was there consistency between the data presented and the ﬁndings? | Relationship to existing knowledge |  |
| 31. Clarity of major themes | Were major themes clearly presented in the ﬁndings? | Results |  |
| 32. Clarity of minor themes | Is there a description of diverse cases or discussion of minor themes? | Discussion |  |

**CHECKLIST OF STANDARDS FOR REPORTING QUALITATIVE RESEARCH (SRQR)**

| **Item No.** | **Topic** | **Standard SRQR description** | **Page No.** | **How the manuscript adheres to the reporting standards** |
| --- | --- | --- | --- | --- |
| **Title and abstract** | | | | |
| S1 | Title | Concise description of the nature and topic of the study Identifying the study as qualitative or indicating the approach (e.g., ethnography, grounded theory) or data collection methods (e.g., interview, focus group) is recommended | 1 | We describe the nature and topic of the study. |
| S2 | Abstract | Summary of key elements of the study using the abstract format of the intended publication; typically includes background, purpose, methods, results, and conclusions | 2-3 | We summarize key elements of the study using the abstract format. |
| **Background** | | | | |
| S3 | Problem formulation | Description and significance of the problem/phenomenon studied; review of relevant theory and empirical work; problem statement | 4-5 | We describe both our problem studied and a review of relevant literature. |
| S4 | Purpose or research question | Purpose of the study and specific objectives or questions | 5 | We describe the purpose of the study and specific research objectives. |
| **Methods** | | | | |
| S5 | Qualitative approach and research paradigm | Qualitative approach (e.g., ethnography, grounded theory, case study, phenomenology, narrative research) and guiding theory if appropriate; identifying the research paradigm (e.g., postpositivist, constructivist/interpretivist) is also recommended; rationale | 5 | In the background, we describe the COM-B model and the BCW framework as our methodological orientation and guiding theory. |
| S6 | Researcher characteristics and reflexivity | Researchers’ characteristics that may influence the research, including personal attributes, qualifications/experience, relationship with participants, assumptions, and/or presuppositions; potential or actual interaction between researchers’ characteristics and the research questions, approach, methods, results, and/or transferability | 5-8 | We report that the interviewers did not know the study participants prior to study commencement.  We also report that the participants were informed of the goal of this formative study; which was to inform the design of an implementation of integrated COVID-19 and TB screening, which was to be conducted at the same hospital.  We do not describe interviewer characteristics like bias, assumptions and others. |
| S7 | Context | Setting/site and salient contextual factors; rationale | 6 | We describe where and how interviews were conducted. |
| S8 | Sampling strategy | How and why research participants, documents, or events were selected; criteria for deciding when no further sampling was necessary (e.g., sampling saturation); rationale | 5-8 | We describe the purposive sampling technique we used to select participants, how we approached the participants and the sample size. We also describe how we arrived at data saturation. |
| S9 | Ethical issues pertaining to human subjects | Documentation of approval by an appropriate ethics review board and participant consent, or explanation for lack thereof; other confidentiality and data security issues | 6-7 | We describe that verbal informed consent was obtained from all participants prior to the interviews.  We describe our data management procedures including security and that data is de-identified.  We report that this study received ethical approvals received ethical approvals from the AIDS support organization (TASO) research and Ethics Committee (TASO REC 082/2020-UG-REC-009) and the Uganda National Council of Science and Technology (HS1152ES). |
| S10 | Data collection methods | Types of data collected; details of data collection procedures including (as appropriate) start and stop dates of data collection and analysis, iterative process, triangulation of sources/methods, and modification of procedures in response to evolving study findings; rationale | 6-7 | We describe the data collection methods (semi-structured interviews) and the start and stop dates of data collection (between January 2021 and March 2021). |
| S11 | Data collection instruments and technologies | Description of instruments (e.g., interview guides, questionnaires) and devices (e.g., audio recorders) used for data collection; if/how the instrument(s) changed over the course of the study | 6-7 | We describe interview guides including topics and questions.  We report that we used audio recorders to record the interviews. |
| S12 | Units of study | Number and relevant characteristics of participants, documents, or events included in the study; level of participation (could be reported in results) | 9 | We describe the number (12 healthcare Providers) and relevant characteristics of participants in the results. |
| S13 | Data processing | Methods for processing data prior to and during analysis, including transcription, data entry, data management and security, verification of data integrity, data coding, and anonymization/ deidentification of excerpts | 8-9 | We report that audio interviews were transcribed verbatim. We describe how the transcripts were de-identified and secured and the process of data coding by two analysts. |
| S14 | Data analysis | Process by which inferences, themes, etc., were identified and developed, including the researchers involved in data analysis; usually references a specific paradigm or approach; rationale | 7-9 | We describe how themes were derived from the data using an inductive approach and the researchers involved.  We describe the application of the COM-B model and BCW framework to the emergent themes. |
| S15 | Techniques to enhance trustworthiness | Techniques to enhance trustworthiness and credibility of data analysis (e.g., member checking, audit trail, triangulation); rationale | 8 | We describe the processes of investigator triangulation and peer debriefing. |
| **Results/findings** | | | |  |
| S16 | Synthesis and interpretation | Main findings (e.g., interpretations, inferences, and themes); might include development of a theory or model, or integration with prior research or theory | 9-13 | We describe the main findings of the study and integrate them into the COM-B model and BCW framework. |
| S17 | Links to empirical data | Evidence (e.g., quotes, field notes, text excerpts, photographs) to substantiate analytic findings | 9-13 | Quotations are presented to substantiate the analytical findings. We believe that the results are clearly presented. |
| **Discussion** | | | |  |
| S18 | Integration with prior work, implications,  transferability, and contribution(s) to the field | Short summary of main findings; explanation of how findings and conclusions connect to, support, elaborate on, or challenge conclusions of earlier scholarship; discussion of scope of application/ generalizability; identification of unique contribution(s) to scholarship in a discipline or field | 13-16 | We discuss our main findings within the context of the existing literature and identify our unique contributions.  We offer implications for future research and practice. |
| S19 | Limitations | Trustworthiness and limitations of findings | 15-16 | We describe both study strengths and limitations. |
| **Other** | | | |  |
| S20 | Conflicts of interest | Potential sources of influence or perceived influence on study conduct and conclusions; how these were managed. | 24 | We declare no conflicts of interest. |
| S21 | Funding | Sources of funding and other support; role of funders in data collection, interpretation, and reporting. | 24 | We declare that the study supported by a grant from the Government of Uganda through the Makerere University Research and Innovation Fund (Mak RIF). The funders had no role in the design, collection, analysis, and interpretation of data; in the writing of the manuscript; and in the decision to submit this manuscript for publication. |
